# Dorsal Posterior Parietal Cortex Lesions Disrupt Spatial‐ but Not Motor‐Based Inhibition

**DOI:** 10.1111/ejn.70528

**Published:** 2026-05-06

**Authors:** Julie Ouerfelli‐Ethier, Tristan Jurkiewicz, Isabella Comtois‐Bona, Thomas Carrier, Aarlenne Z. Khan, Laure Pisella

**Affiliations:** ^1^ Université Claude Bernard Lyon 1, Centre de Recherche en Neurosciences de Lyon CRNL, INSERM U1028, CNRS UMR5292, Trajectoires, Centre Hospitalier Le Vinatier, Bâtiment 336 Bron France; ^2^ École d'Optométrie Université de Montréal Montréal Québec Canada

**Keywords:** countermanding, inhibition of return, motor intention, saccade planning, spatial attention

## Abstract

Spatial and response inhibition are two different types of inhibition processes. Spatial inhibition refers to the suppression of a specific location, whereas response inhibition involves cancelling a planned movement and is motor based. Here we examined the effects of lesions on the dorsal posterior parietal cortex on performance during two saccade tasks that separately assessed spatial (inhibition of return task) and response inhibition (stop signal task). We tested two stroke patients, one with unilateral and one with bilateral lesions to the dorsal posterior parietal cortex, as well as 21 age‐matched controls. In our spatial inhibition task, control participants showed the typical inhibition of return effect, whereas patients exhibited no inhibition of return in their ataxic hemifields. In contrast, patients and their matched controls performed similarly on the stop signal task. These results reveal a simple dissociation in our patients, where motor‐based inhibition is preserved following damage to the dorsal posterior parietal cortex, whereas spatial inhibition is impaired. This highlights the specific role of the dorsal posterior parietal cortex in spatial inhibition, notably related to spatial attentional mechanisms.

## Graphical abstract

Spatial and response inhibition are different types of inhibition processes that can be separately assessed in two saccade tasks: inhibition of return and stop signal. Optic ataxia patients performed similarly to controls on the stop signal task but did not show normal inhibition of return. This shows that the dorsal posterior parietal cortex is not crucial for response inhibition but underlies the inhibition of a previously explored location in space.

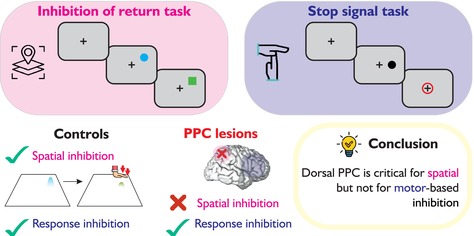

**Abbreviations**
IOR: inhibition of return
IPS: intraparietal sulcus
ITI: inter‐trial interval
PPC: posterior parietal cortex
SD: standard deviation
TPJ: temporo‐parietal junction

## 1 Introduction

Various types of inhibition abilities contribute to the flexibility of human behavior, including spatial and response inhibition. Spatial‐based inhibition refers to the suppression of a specific location in the visual scene. For example, many studies have shown that previously explored or attended locations tend to be suppressed, leading to increases in reaction times for subsequent responses toward this location (Abrams and Dobkin 1994; Briand et al. 2000; Fecteau et al. 2004; Fecteau and Munoz 2005; Hunt and Kingstone 2003; Klein 2000; Posner et al. 1985; Posner and Cohen 1984; Pratt and Neggers 2008; Rafal et al. 1994; Sapir et al. 2014). This phenomenon, classically labelled as inhibition of return (IOR) (Klein 2000; Posner et al. 1985; Posner and Cohen 1984), facilitates orienting toward novel and previously unexplored locations (Chica et al. 2010; Kingstone and Pratt 1999; Klein and MacInnes 1999; Posner et al. 1985; Rafal et al. 1989; Ro et al. 2003). In contrast, response inhibition is motor‐based and involves cancelling a planned movement due to changing contextual demands (Logan 1983; Logan and Cowan 1984). This type of inhibition is commonly assessed using stop signal or countermanding paradigms (Verbruggen and Logan 2008) in which participants make frequent and speeded motor responses, but they must be prepared to inhibit their response when a stop signal is presented. Spatial and response inhibition can mainly be differentiated along two dimensions. First, they differ in their selectivity: spatial inhibition relies on a selective and spatially specific mechanism where one precise location is suppressed. In contrast, response inhibition relies on a global, non‐specific mechanism where a prepotent action is cancelled irrespective of its specific spatial goal and the location and sensory modality of the stop signal (Asrress and Carpenter 2001; Aron and Verbruggen 2008); it is also invariant to spatial interference (Logan 1981; van den Wildenberg and van der Molen 2004). Second, at a conceptual level, the type of competition underlying spatial‐based inhibition and response inhibition also differs; during spatial‐based inhibition, locations automatically compete for *attentional* selection, whereas response inhibition pertains to control of motor initiation and behavioral mechanisms of executive functions.

The neuroanatomical basis differentiating spatial and response inhibition remains unclear, particularly with respect to the involvement of the parietal cortex. In monkeys, spatial‐based inhibition has been primarily associated with activation in the lateral intraparietal area (Bisley and Goldberg 2010; Bisley and Mirpour 2019; Goldberg et al. 2006; Ptak 2012; Sprague and Serences 2013). In humans, it has been shown that spatial inhibitory mechanisms are impaired following lesions to the inferior parietal lobule; that is, the ventral part of the posterior parietal cortex (PPC). For example, patients with such lesions, both with and without hemineglect, show impaired (i.e., facilitatory instead of inhibitory, or mislocated) IOR in their ipsilesional hemifield (Bartolomeo et al. 1999, 2001; Bourgeois et al. 2012; Sapir et al. 2004; Vivas et al. 2003, 2006, 2008), consistent with behavior such as spatial scanning disorganization or increased returns to previously visited locations (Pisella and Mattingley 2004). Recent transcranial magnetic stimulation (TMS) findings have revealed complex patterns of IOR impairments following right stimulation to the intraparietal sulcus (IPS) and temporo‐parietal junction (TPJ) with distinctive hemifield and task effects (Bourgeois et al. 2013a). Specifically, for right‐sided targets (i.e., ipsilateral stimulation), manual IOR was impaired, whereas saccadic IOR was preserved when either the IPS or the TPJ were stimulated. For left‐sided targets (i.e., contralateral stimulation), TMS on the IPS impaired both manual and saccadic IOR, whereas TMS on TPJ had no effect. As such, the dorsal and ventral parts of the PPC, and the TPJ, may play a distinct role in spatial‐based inhibition.

Concerning response inhibition, neuroimaging studies have demonstrated increased activation in both dorsal and ventral PPC regions during Go/NoGo and stop signal tasks (Aron and Poldrack 2006; Congdon et al. 2010; Hu and Li 2012; Jaffard et al. 2008; Kelly et al. 2004; Menon et al. 2001). A TMS study showed that applying stimulation to the IPS, but not to the TPJ, prolonged the time necessary for participants to cancel their motor plan, according to functional connectivity between IPS and both the inferior frontal cortex and the presupplementary motor area (Osada et al. 2019). Another TMS study found no effect of ventral PPC stimulation during a stop signal task (Y. Cai et al. 2016). However, patients with left spatial neglect have been shown to have a bilateral, non‐spatially specific increase in the rate of incorrect reflexive saccades toward the visual target as well as increased latencies of anti‐saccades (Butler et al. 2009), findings that are consistent with a response inhibition deficit.

These findings taken together, the involvement of the PPC in both spatial and response inhibition remains unclear. Recent work from our group testing patients with optic ataxia in an anti‐saccade task suggested a crucial role of the dorsal PPC for spatial inhibition only (Ouerfelli‐Ethier et al. 2021, 2023). Optic ataxia is a rare neurological disorder caused by lesions to the dorsal PPC, which is mainly associated with inaccurate visually‐guided arm movements toward the contralesional hemifield in the absence of primary visual and motor impairments (Pisella et al. 2021). During anti‐saccades, participants have to direct a saccade in the opposite direction of a visual target. Spatial miscalculations of the saccadic goal were biased toward the visual stimulus location where spatial inhibition should have occurred (Ouerfelli‐Ethier et al. 2021, 2023); these results are consistent with spatial‐based inhibition deficits, rather than response inhibition deficits. Anti‐saccades directed to the ipsilesional field were also delayed in unilateral lesions to the dorsal PPC (Ouerfelli‐Ethier et al. 2023). In our opinion, this is also in line with decreased spatial suppression of the visual target location in the contralesional field (Ouerfelli‐Ethier et al. 2021, 2023). However, given the anti‐saccade task does not allow us to conclusively distinguish between spatial and response inhibition processes, it is difficult to determine the role of the dorsal PPC in each inhibitory process from these results alone.

To refine our understanding of the role of the dorsal PPC in both spatial‐ and motor‐based inhibition, we assessed two stroke patients with optic ataxia on tasks measuring spatial and response inhibition separately: an IOR task measuring spatial‐based inhibition, a stop signal task measuring response inhibition, and a control visually‐guided saccade task requiring no inhibitory mechanisms. For the IOR task, we speculated that if our patients have spatial‐based inhibition impairments, they should show an absent or reduced IOR relative to controls in their affected hemifields; that is, for our lesioned patient with left optic ataxia, we would expect impaired IOR in their left hemifield (i.e., left cue and target) and intact IOR in their right hemifield (i.e., right cue and target). In the stop signal task, impaired motor inhibition would lead to higher failed stop frequencies in the patients relative to their controls, with possible asymmetries for the unilateral optic ataxia patient between leftward and rightward saccades. We further manipulated the proportion of stop signals within a block. When stop signals were presented infrequently (20% stop signal ratio), participants' performance is likely biased toward the “go” process, leading to seemingly more impulsive responses due to reduced proactive preparation to stop. Conversely, at a higher stop signal ratio (40%), participants tend to be more prepared to stop and thus more biased toward inhibition (Friedrich et al. 1998; Logan and Cowan 1984; Ramautar et al. 2004, 2006; van den Wildenberg et al. 2002). If the dorsal PPC contributes to flexible inhibitory control mechanisms involved in response inhibition, we would expect that the patients would show increased difficulty at the infrequent (20%) ratio (i.e., higher SSRTs) compared with the frequent (40%) (i.e., lower SSRTs), and this difference between ratios would differ from controls. Finally, to rule out the possibility that any impaired performance in our IOR and stop signal task were due to deficits in saccade planning or execution, we also tested our participants on a control visually‐guided saccade task. If impaired performance in our two previous tasks is solely the result of impaired inhibitory mechanisms, saccade metrics in this control task should not differ between our patients and their controls.

## 2 Materials and Methods

### 2.1 Participants

We recruited a total of 23 participants. This included two patients with strokes involving a common lesion to the dorsal PPC (Brodmann's area 7) leading to optic ataxia. Patient C.F. showed unilateral optic ataxia in his left hemifield and was tested along with 11 age‐matched controls. Patient I.G. showed bilateral optic ataxia and was tested along with 10 age‐matched controls. We confirmed that our two patients and their respective controls were age‐matched with one‐sample *t*‐tests (see Table 1). All participants had normal or corrected‐to‐normal vision.

**TABLE 1:** Mean age and age comparisons between patients with optic ataxia and age‐matched participants.

Groups	*N*	Mean (y)	SD (y)	Range	*t*	*p*
IG	1	53				
IG's controls	10	52.4	3.27	46–56	−0.580	0.576
CF	1	45				
CF's controls	11	44.09	1.92	41–47	−1.569	0.148

Abbreviations: *p*, *p*‐values; SD, standard deviation; *t*, one‐sample *t*‐tests.

**FIGURE 1:**
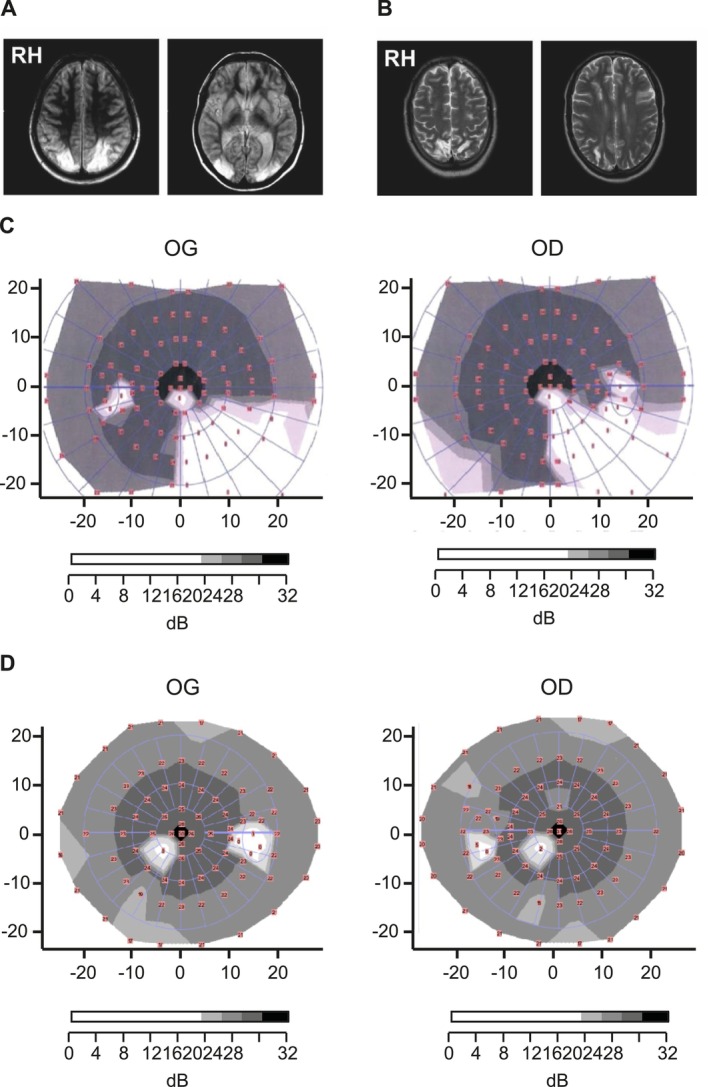
Neurological and visual field assessment of patients I.G. and C.F. In (A), T1 anatomical scan of I.G.'s brain shows bilateral dorsal posterior parietal and occipital lesions. In (B), we present C.F.'s asymmetrical bilateral lesions in the dorsal posterior parietal cortex where damage is larger in the right hemisphere in T1 anatomical scan. RH, right hemisphere. Visual assessment was performed with Fiber Adapted Static Testing (F.A.S.T.) perimetry: In (C), we present I.G.'s lower right quadrantanopia in both eyes and in (D), C.F.'s lower left scotoma in both eyes. OD: oculus dexter or right eye, OG: oculus sinister or left eye.

Patient I.G. is a right‐handed 53‐year‐old female who suffered an ischemic stroke 28 years prior to testing, related to acute vasospastic angiopathy which caused the blockage of the posterior cerebral arteries. Her lesions were mostly limited to the dorsal PPC (Brodmann's areas 7 and 39; Figure 1A) symmetrically in both hemispheres leading to chronic bilateral optic ataxia, her visually guided movements being inaccurate bilaterally in her peripheral vision (Gaveau et al. 2008; Jurkiewicz et al. 2025; McIntosh et al. 2011; Pisella et al. 2000). The lesion also included some damage to the superior part of occipital Brodmann's areas 18 and 19. I.G. shows no primary motor, somatosensory or visual deficits except for a quadrantanopia in the lower right visual field, due to damage to the optical fibers passing below the parietal cortex (Pisella et al. 2000) (see Figure 1C).

Patient C.F. is a right‐handed 45‐year‐old male who suffered from a watershed posterior infarct 22 years prior to testing, which resulted in asymmetrical bilateral lesions of the dorsal PPC (Brodmann's areas 7 and 5; Figure 1B.) Larger damage to BA 7 in the right hemisphere led to chronic unilateral optic ataxia in the left hemifield (Blangero et al. 2008; Jurkiewicz et al. 2025; Khan, Pisella, Vighetto, et al. 2005). He also displayed some damage to the median occipital gyrus (BA 18 and 19) and to Brodmann's area 2 with a minute extension to the semiovale centers. However, symptoms following his lesions were specific to visually‐guided movements, and he otherwise did not show any purely motor or somatosensory impairment. He also had no purely visual deficits, except for a small and localized scotoma in the lower left quadrant that was discovered accidentally during the present testing (see Figure 1D). Recent clinical tests performed with optical coherence topography imaging showed both normal macula thickness and normal optical nerve thickness across quadrants. The thickness of the ganglion cells layer was also assessed as normal and symmetrical between both eyes.

Procedures received ethics approval from the ethical committee for clinical research (CERC) at the University of Montreal. Procedures in Lyon conformed to the French law (March 4, 2002) on human subjects' rights and informed written consent was collected from all participants in accordance with CPP South Mediterranean III with the registration number 2019‐A03055‐52. We observed the privacy rights of our participants in accordance with our ethics protocols in both Montreal and Lyon. Participants received financial compensation upon completion.

### 2.2 Apparatus

Testing occurred conjointly at the University of Montreal (Montreal, Canada) and Centre of Neuroscience research of Lyon (Lyon, France) on similar setups. Participants sat 57 cm away from a high‐speed computer screen (in Montreal: 20.5*12 in., ViewSonic XG2405, ViewSonic Corporation, Brea, California, United States; in Lyon: 20.5*12 in., BenQ XL2420, BenQ Corporation, Taipei city, Taipei, Taiwan) in a dark room. On both setups, chin and forehead rests restricted movement during the tasks, whereas an eye‐tracker recorded eye movements (EyeLink 1000 Plus, SR Research, Kanata, Canada, frequency: 1000 Hz).

### 2.3 Procedure

**FIGURE 2:**
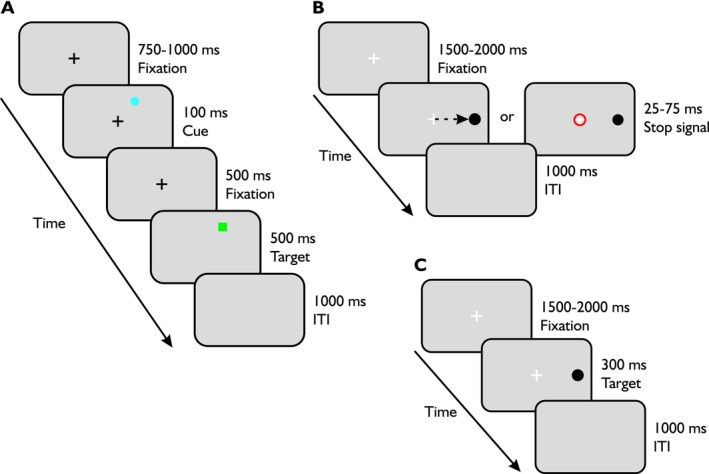
Experiment sequence and timings of the tasks. The IOR task is shown in (A). Each trial started with a central black fixation cross. Next, a cue (i.e., a blue circle) appeared briefly in one of the four corners of the screen; participants were instructed to ignore it and maintain their fixation on the fixation cross. A green target square randomly appeared after 500 ms at any one of the four locations; it could appear either at the same location as the distractor or elsewhere. Participants were instructed to move their eyes toward the target when it appeared. In (B), we show the experimental sequence for the stop signal task. For most trials, once the target (i.e., black circle) appeared, either on the right or left, participants had to make an eye movement toward it. During some trials, a stop signal was flashed and indicated to the participants that they had to inhibit their response and keep their central fixation. During the visually‐guided saccade task in (C), a fixation cross was presented until a target (i.e., black circle) appeared to the left or to the right. Participants were asked to make a saccade to the target as quickly as possible.

Across two sessions of 1 h each, participants performed three tasks: an exogenous IOR task (see Figure 2A), a stop signal task (see Figure 2B), and a control visually‐guided saccade task (see Figure 2C). The order of the tasks was randomized across participants. For each of the tasks, participants first completed a practice block to familiarize themselves with the task and to ensure they understood the experimenter's instructions.

Each task was divided in short blocks lasting no more than 5 min, allowing participants to take breaks between blocks and tasks to minimize fatigue. The IOR task comprised three blocks of 35 trials. For the stop signal task, we tested participants on separate blocks of 20% and 40% stop signal ratios and the number of blocks varied per participant depending on whether each block was assessed as correct on‐line (i.e., not violating the assumption of independence; see Section 2.4 for a detailed description of how this was established on‐ and off‐line). The 20% ratio blocks had 60 trials, whereas the 40% ratio blocks had 45 trials to obtain similar numbers of stop trials. The control visually‐guided saccade task consisted of a single block of 40 trials.

All tasks were designed and implemented using MATLAB (The MathWorks Inc., Natick, Massachusetts, United States) with the Psychophysics toolbox (Brainard 1997).

#### 2.3.1 IOR Task

Participants were instructed to ignore a flashed cue (blue circle) and to only make a saccade toward a target (green square) when it appeared. The task was set against a pale grey background. At the beginning of each trial, participants maintained their fixation on a black centered cross of 1° diameter for a duration randomized between 750 and 1000 ms. After the fixation screen, a blue circle cue (diameter: 1.5°) appeared for 100 ms. After its disappearance, the fixation cross remained alone for 500 ms before a green square (diameter: 1.5°) appeared for 500 ms. There were four possible locations for both the target and cue. Those locations were 8° equidistant from the fixation cross and 90° angular away from each other, starting at a position of 45° angular from the horizontal meridian. The positions of the target and cue were randomized across possible locations; thus the target appeared at the same location as the cue for 25% of trials containing a cue (totalling 20% of overall trials), and elsewhere for 75% of those trials (60% of overall trials). For the remaining 20% of the trials, no cue was presented before the target; these trials were uncued trials. We informed participants that the blue circle cue was not important for the task and not predictive of where the target could appear. The interval inter‐trial lasted 1000 ms. For our two optic ataxia patients, we modified the task so that neither target nor cues appeared in the lower left quadrant for C.F. or in the lower right quadrant for I.G.

#### 2.3.2 Stop Signal Task

Participants were asked to make a saccade toward a target, except when a red circle surrounded the fixation, in which case they were required to try and maintain fixation. Participants fixated on a centered white fixation cross (diameter: 0.5°) set against a pale grey background for a randomized duration between 1500 and 2000 ms. A dark grey circle (0.75° of diameter) then appeared at 5° eccentricity on the horizontal meridian, either to the right or to the left. The target remained visible for 300 ms. For trials with a stop signal, an empty red circle (thickness: 5 px; diameter: 0.66°) appeared for 500 ms around the fixation cross which indicated to participants to withhold their saccade. The delay between the appearance of the target and stop signal varied based on a staircase algorithm applied to each participant and block of trials. During stop signal trials, participants' responses were classified on‐line as either a successful or failed stop. Failed stop trials were those where a saccade to the target was detected, with eye position landing within 3° of the visual target's location, whereas trials where eye position remained outside this 3° zone were classified as successful stops. The stop signal delay, the time between target and stop signal onset, increased by 25 ms following a successful stop and decreased by 25 ms following a failed stop. The online adjustment was used to estimate participants' performance and to adjust the stop‐signal delay (SSD) in order to maintain an overall proportion of approximately 50% successful and 50% failed stop trials. Participants often interpreted failed stop trials as errors or failures; they were therefore explicitly informed that successful stopping on only about 50% of stop trials was expected and necessary for estimating their stopping threshold. They were informed that the goal was to identify the threshold at which stopping was successful on half of the stop trials, and that it was normal to be unable to withhold their saccade on every stop‐signal trial. To discourage strategic slowing, there was an auditory cue if the eye position was not detected within 3° of the visual target's location on go (no stop) trials after 325 ms. At the end of each trial, there was an ITI of 1000 ms.

#### 2.3.3 Control Visually Guided Saccade Task

This task served as a control “non‐inhibitory” task during which participants were instructed to make a saccade toward the target as quickly as possible. At the beginning of each trial, participants maintained their gaze on a white centered fixation cross (diameter: 0.5°) set against a pale grey background for a randomized duration between 1500 and 2000 ms. Thereafter, the target (i.e., dark grey circle of 0.75° of diameter) appear on the right or on the left, 5° away from the central fixation cross on the horizontal meridian and it remained on for 300 ms. This screen was followed by a blank ITI of 1000 ms.

### 2.4 Preliminary Analysis

We recorded a total of the 14,400 trials, which corresponded to 2510 trials for the IOR task, 11,005 for the stop signal task, and 885 for the control visually‐guided saccade task. We first visually inspected each trial for each participant to ensure the quality of the recording. Due to the poor quality of the recording, one control for I.G. was excluded from further analysis; they had too few trials for any behavioral measures to be assessed for each task. We next conducted separate preliminary analyses for each task as described below. We only considered the first saccade for each trial made by participants.

#### 2.4.1 IOR Task

After visual inspection of each trial, we removed all trials where eye signal was lost during either cue or target presentation because of blinks, participants moving or technical issues (114 trials, 4.54% of IOR trials). Considering patients were tested on modified versions of the task where the target nor the cue were presented on the bottom right for I.G. and on the bottom left for C.F., we also removed those respective trials to normalize saccade sampling locations across participants (900 trials, 35.86% of IOR trials). In other words, we excluded all trials where either the cue or the target appeared in the bottom right quadrant for I.G.'s controls, and in the bottom left quadrant for C.F.'s controls, thus only keeping trials for possible cue and target locations for each respective patient. Consequently, this led to unbalanced number of trials across the four potential target locations in the IOR task. For I.G. and her control subjects, the solution to circumvent this limitation was to analyze trials for only the upper visual field for the IOR effects. For C.F. and his control subjects, because we aimed at contrasting data between hemifields, we additionally removed all invalid trials during which the cue and the target appeared in the right hemifield (106 trials, 4.22% of IOR trials) that could not be matched to similar invalid trials within the left hemifield. This allowed us to statistically test comparable conditions of valid cueing (IOR) and comparable conditions of invalid cueing across hemifields, with the target presented in the left versus the right side.

We calculated saccade reaction times (SRTs) for the first saccade by subtracting saccade onset from target onset. We retained only trials in which the SRT was between 80 and 1000 ms; these saccade latencies outbounds were established to remove anticipatory or overly delayed responses (one trial, 0.04% of total number of IOR trials). We also calculated saccade amplitude and absolute directional error relative to the target for correct trials. We removed all trials where saccade amplitude was below 2° or above 20° or the absolute directional error was beyond 40° angular on either direction of the visual target (two trials, 0.08% of total number of IOR trials). Saccades to the cue or elsewhere were thus discarded and not used in any behavioral analyses. For subsequent analyses, there remained 1387 trials, which corresponded to 55.26% of total IOR trials.

We sorted the remaining trials according to target location relative to cue location: when the target appeared at the same location as the cue (valid trials) versus when the target appeared elsewhere (invalid trials). We calculated IOR effects per participant by subtracting mean SRTs for invalid trials from mean SRTs for valid trials. Positive numbers indicated the presence of an IOR effect (i.e., participants' SRTs were longer when the cue and the target appeared at the same location), whereas negative numbers indicated a facilitation effect (i.e., participants' SRTs were shorter when the target reappeared at the location previously occupied by the cue).

#### 2.4.2 Stop Signal Task

From the total of trials recorded, we removed all trials where eye signal was lost during target presentation as a result of blinks or participants moving by visually inspecting each trial (111 trials, 1.09% of stop signal trials). We classified every trial for the stop signal blocks as either successful go, failed go, successful stop, or failed stop. We defined successful go trials as all trials without a stop signal with saccade amplitudes ranging between 2.5° and 15° amplitude, an SRT of between 80 and 1000, and within 45° angular of target position. Failed go trials were trials without a stop signal but without a saccade or with a saccade that had an amplitude lower than 2.5°, or an SRT shorter than 80 ms or longer than 1000 ms. Successful stop signal trials were defined as all trials with a stop signal and without a saccade or with a saccade smaller than 2.5° or with an SRT longer than 1000 ms. Stop signal trials were considered failed when a saccade of at least 2.5° amplitude with an SRT of between 80 and 1000 ms was directed within 45° angular of target location. Trials that could not be classified as either successful go, failed go, successful stop, or failed stop were discarded (11 trials, 0.10% of stop signal trials).

We calculated the stop signal reaction times (SSRTs), as our measure of performance for the stop signal task, which is an estimate of the speed of the stop process or the speed at which a motor plan is successfully cancelled (Allen et al. 2018; Boucher et al. 2007; Logan and Cowan 1984; Verbruggen et al. 2019). SSRT was estimated using the integration method by subtracting the starting time of the stop process from its finishing time (Band et al. 2003; Logan 1994; Logan and Cowan 1984; Verbruggen et al. 2019). The starting time of the stop process corresponds to the mean stop signal delay (see the description of the stop signal task above for a description of how the stop signal delays were obtained). As for the finishing time of the stop process, it is calculated by integrating the go trials SRT distribution from the onset of the target until the resulting cumulative proportion corresponds to the observed proportion of failed stop trials. In other terms, if the proportion of failed stop was 50% for a participant, the finishing time of the stop process would average around the 50th percentile of the go SRTs distribution. To obtain a reliable measure of response inhibition, we used a replacement method where all failed go trials (where no saccades were executed) were integrated to the go SRTs distribution: the maximal successful go trials reaction times per participant and per ratio were attributed to them (Band et al. 2003; Verbruggen et al. 2019). For the estimation of SSRTs to be reliable, preliminary per‐block analyses were conducted following the approach described in Verbruggen et al. (2019). Their approach preserves the race model's key assumption that the go and stop processes operate independently (Boucher et al. 2007; Logan and Cowan 1984). Accordingly, we removed all blocks where the percentage of failed go was above 10%. We also only considered blocks where the percentage of failed stop was between 25% and 75%, and we confirmed that the mean failed stop SRT was numerically shorter than the mean go SRT. We then estimated SSRT per block, and we removed all blocks with SSRTs below 50 ms (Congdon et al. 2012). In total, we removed 18 blocks: 12 blocks for the 20% stop signal ratio and six blocks for the 40% stop signal ratio (940 trials, 8.54% of stop signal trials). There remained a total of 10,065 trials for subsequent analyses (91.46% of stop signal trials).

#### 2.4.3 Control Visually‐Guided Saccade Task

For this task, we also visually inspected each trial and removed all trials where the eye signal was lost during target presentation (six trials, 0.68% of control task trials). We calculated SRTs for the first saccade recorded relative to target onset. We removed all trials with SRTs below 80 ms and beyond 1000 ms, with small (i.e., below 2.5°) or large amplitudes (i.e., 15°) or directed beyond 45° angular from target direction (one trial, 0.11% of control task trials). There remained 878 trials for subsequent analyses.

Statistical analyses were conducted on MATLAB R2024b (The MathWorks Inc., Natick, Massachusetts, United States). To compare our optic ataxia patients with their respective controls, we used modified *t*‐tests developed specifically for single cases in neuropsychology (Crawford et al. 2010; Crawford and Garthwaite 2002, 2005; Crawford and Howell 1998) based on MATLAB scripts by Schrooten (2017) obtained from MATLAB File Exchange. Because of the lower statistical power inherent to single case studies, these analyses recommend at least six controls per single case, a number we surpassed for both our control groups: I.G. had nine age‐matched controls, whereas C.F. had 11.

## 3 Results

For the IOR task, we first reported SRTs per trial type for our patients and their respective controls. We next tested the presence of an IOR effect in our patients and their control groups. These statistical analyses were followed by comparing the magnitude of the IOR effect between each patient and their respective control group.

For the stop signal task, we examined SSRT across ratios of stop signal and participant groups, as well as mean SRTs across trial type (i.e., successful go, successful stop, and failed stop).

For the control visually‐guided saccade task, we compared SRTs between each patient and their respective controls. Given C.F.'s unilateral optic ataxia, we also tested the difference in SRTs between rightward and leftward SRTs between this patient and his controls.

### 3.1 IOR Task

**FIGURE 3:**
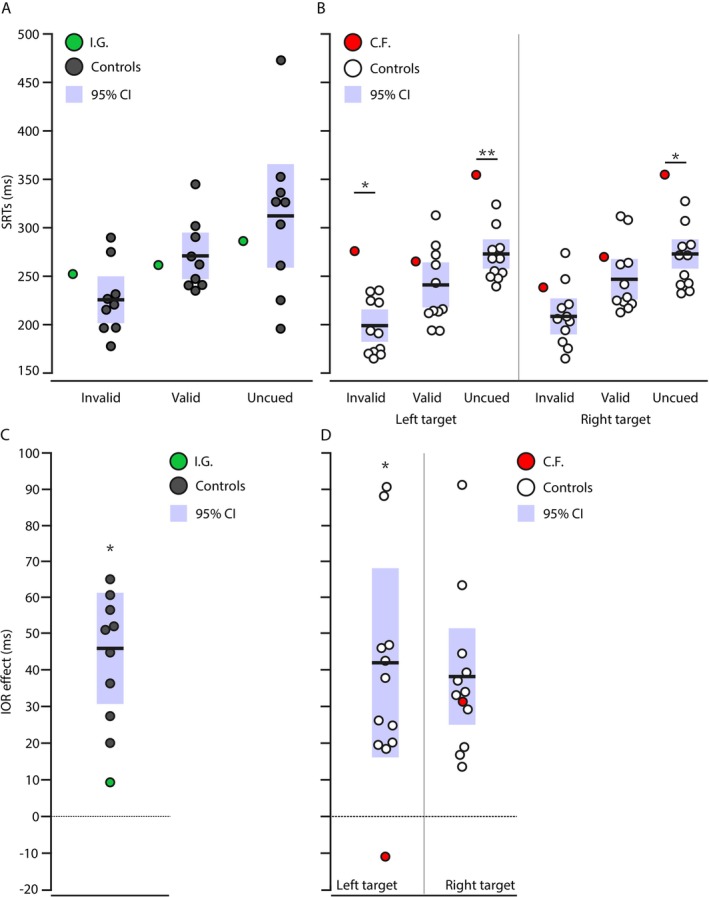
Behavioral measures for the IOR task. In all panels, I.G. appears in green, with her controls in dark grey, whereas C.F.'s performance is presented in red, and his controls in white. For C.F. and his controls, performance is reported separately for left and right targets. In panels (A, B), we show overall SRTs per trial type for each patient and their respective control group. Invalid trials refer to different cue/target locations trials, whereas valid trials are same cue/target location trials. In (C, D), mean IOR effects are presented across participants as individual datapoints. A bold horizontal line indicates mean IOR effects across participants and shaded areas are 95% confidence intervals. As shown on the y‐axis, positive IOR effects quantify the inhibitory effect at the valid target location. In contrast, negative IOR effects mark a facilitation effect for saccades directed at the valid target location. * = *p* < 0.05; ** = *p* < 0.01.

Mean SRTs for different cue/target locations (i.e., invalid), same cue/target locations (i.e., valid), and uncued trials are depicted in Figure 3A,B for all our participant groups; SRTs for I.G. are depicted in green alongside her controls in dark grey, whereas C.F.'s SRTs are shown in red alongside his controls in white. With modified *t*‐tests, we compared mean SRTs for each trial type between each patient and their controls. For I.G., we found no significant difference between our patient and her controls for all trial types, suggesting overall SRTs were not affected in our bilateral patient for this task relative to controls: invalid locations, *t*(8) = 0.6818, *p* = 0.2573; valid locations, *t*(8) = −0.2448, *p* = 0.4064; uncued trials, *t*(8) = −0.3012, *p* = 0.3855 (Figure 3A). As for C.F., we tested each side of the hemifield separately because of his unilateral left optic ataxia. C.F.'s SRTs for invalid locations were increased compared with controls for left targets, *t*(10) = 2.6229, *p* = 0.0127, but not for right targets, *t*(10) = 0.9108, *p* = 0.1919. C.F.'s SRTs for valid location trials did not differ from controls for either left targets, *t*(10) = 0.6, *p* = 0.2813, and right targets, *t*(10) = 0.6225, *p* = 0.2738. For uncued trials, C.F.'s SRTs were longer for both left, *t*(10) = 3.0553, *p* = 0.0061, and right targets, *t*(10) = 2.2345, *p* = 0.0241, compared with his controls (Figure 3B).

For the remaining of our analyses, we calculated IOR effects by subtracting SRTs for invalid locations from SRTs for valid locations. I.G.'s IOR effect is illustrated in green (Figure 3C), whereas C.F.'s IOR effect is in red (Figure 3D). Controls' mean is represented by a black line and 95% confidence intervals by a shaded grey area. IOR effects were numerically greater for controls (I.G.'s controls: M = 45.07, SD = 14.86; C.F.'s controls left targets: M = 41.89 ms, SD = 25.65 ms; C.F.'s controls right targets: M = 38.22 ms, SD = 22.28 ms) than for our two patients. I.G. showed a small IOR effect (M = 9.24 ms). As for C.F., we found a facilitatory effect for left targets appearing at a previously cued location (negative IOR effect M = −10.65 ms), and a regular IOR effect for saccades made to targets presented in the right hemifield (M = 31.35 ms). We compared the magnitude of the IOR effect between each patient and their controls with modified *t*‐tests. I.G.'s IOR effect was statistically lower compared with her controls, *t*(8) = −2.2874, *p* = 0.0258. The same significant result was found with only the upper visual field, although most participants have a total of 10 trials for the valid trial type: *t*(8) = −1.8978, *p* = 0.0471. As for C.F., we found a significantly lower IOR effect for left targets, *t*(10) = −1.9611, *p* = 0.0391, whereas there was no significant difference for right targets, *t*(10) = −0.2952, *p* = 0.3869.

In sum, we found an IOR effect in control participants, but not in our optic ataxia patients, I.G. and C.F., for their respective ataxic hemifields.

### 3.2 Stop Signal Task

**FIGURE 4:**
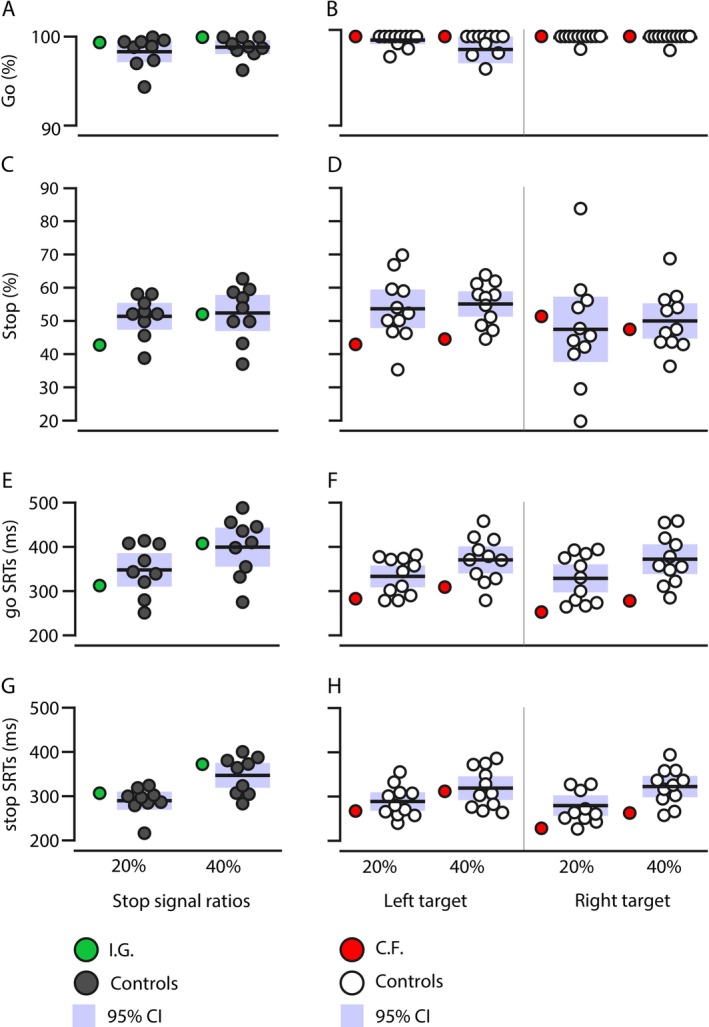
Stop signal task behavioral measures. We show in left panels, task performance for I.G. and her controls. C.F. and his controls' performance are in the right panels, separated by target side. (A, B) Mean percentage of successful go; in (C, D), mean percentage of failed stop; in (E, F), go SRTs; and in (G, H), failed stop SRTs. Of note, the y‐axis in panels (A–D) differs between go and stop trials: in panels (A, B) (go trials), percentages range from 95% to 100%, whereas in panels (C, D) (stop trials), they vary between 30% and 60%. These measures are showed per stop signal ratio and follow the same color code as in previous figures: I.G. and her controls are illustrated in green and in dark grey, respectively. C.F.'s results are in red and his controls in white. Shaded areas represent 95% confidence intervals.

In Figure 4, we illustrate behavioral performance for go and stop trials. First, we compared differences in go trials with percentage of successful go responses (i.e., saccade toward the target) and SRTs for go responses separately for 20% and 40% stop ratios. Low percentage of failed go trials point to participants anticipating a stop signal, which can affect the estimation of SSRTs (Verbruggen et al. 2019). For percentage of successful go during 20% stop ratios, we found no significant difference between I.G. and her controls, *t*(8) = 0.4743, *p* = 0.324, and, between C.F. and his controls for both left, *t*(10) = 0, *p* = 0.500 and right targets, *t*(10) = 0, *p* = 0.5. For the 40% stop signal ratio, percentage of successful go also did not differ from age‐matched controls and for I.G., *t*(8) = 0.959, *p* = 0.185 and for C.F. for both left *t*(10) = 0.319, *p* = 0.378 and right targets, *t*(10) = 0, *p* = 0.500. Participants had overall perfect or near‐perfect mean percentage of successful go trials, as seen in Figure 4A, indicating that they performed the stop signal task correctly. As for go SRTs, the same pattern of non‐significance emerged for both 20% (I.G.: *t*(8) = −0.5824, *p* = 0.288; C.F. left targets: *t*(10) = −1.159, *p* = 0.137; C.F. right targets: *t*(10) = −1.363) and 40% stop ratios (I.G.: *t*(8) = 0.116, *p* = 0.455; C.F. left targets: *t*(10) = −1.137, *p* = 0.141; C.F. right targets: *t*(10) = −1.585, *p* = 0.072; see Figure 4B).

Next, we tested differences in the percentage of failed stop trials across blocks and their SRTs. As mentioned in Section 2.4, failed stop trials are trials during which a stop signal was presented, but participants did not stop their saccade to the target, which provided us with SRTs. For the failed stop percentage during the 20% stop ratio, we found no significant difference, between I.G. and her controls, *t*(8) = −1.423, *p* = 0.10 as well as between C.F. and his controls for both left, *t*(10) = −1.053, *p* = 0.159 and right targets, *t*(10) = 0.239, *p* = 0.408. I.G. and her controls showed no significant difference for the 40% stop ratio, *t*(8) = −0.119, *p* = 0.454. For left targets, there was no significant difference between C.F.'s mean percentage of failed stop (M = 44%) and his controls (M = 55%, SD = 6%; *t*(10) = −1.755, *p* = 0.055), although we did find a trend toward significance. For right targets, C.F. and his controls did not significantly differ for the 40% stop ratio, *t*(10) = −0.319, *p* = 0.378. As seen in Figure 4C,D, the percentage of failed stop was numerically close to 50% for both the 20% and 40% stop ratios for all our participants. This supports the notion that participants performed the task well and that the resulting SSRTs are reliable (Verbruggen et al. 2019). For failed stop SRTs, we observed non‐significant differences between each patient and their respective controls for both the 20% (I.G.: *t*(8) = 0.512, *p* = 0.311; C.F. left targets: *t*(10) = −0.583, *p* = 0.141; C.F. right targets: *t*(10) = −1.247, *p* = 0.120) and 40% stop ratios (I.G.: *t*(8) = 0.559, *p* = 0.30; C.F. left targets: *t*(10) = −0.152, *p* = 0.441; C.F. right targets: *t*(10) = −1.350, *p* = 0.103; see Figure 4E,F). These first analyses allowed us to confirm good task performance and accurate SSRT estimations.

**FIGURE 5:**
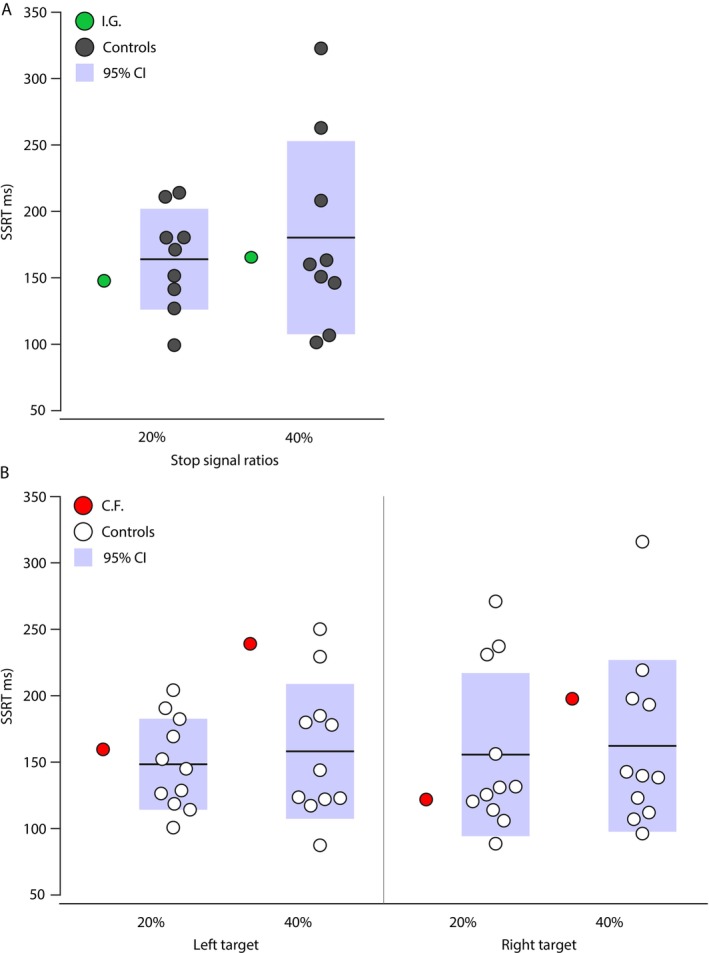
SSRTs across participant groups. Following the same color code as previously, patients and their respective controls' individual datapoints are shown for SSRTs across both stop signal ratios, and per target side for C.F. and his control group. For both control groups, mean SSRTs are marked with thick dark grey lines among the individual datapoints. Shaded areas represent 95% confidence interval.

Finally, we examined differences in SSRTs between each of our patients and their respective controls, separately for 20 and 40% stop signal ratios. We depict in Figure 5 mean SSRTs for I.G. in green (Figure 5A) and her controls in dark grey, whereas C.F.'s SSRTs are in red and his controls in white (Figure 5B). With modified *t*‐tests, we found no significant differences between I.G. and her controls for both the 20%, *t*(8) = −0.449, *p* = 0.333, and the 40% stop signal ratios, *t*(8) = −0.143, *p* = 0.445, nor were there significant differences between C.F. and his controls' SSRTs for either left, *t*_*20%*_(10) = −0.314, *p*_*20%*_ = 0.380; *t*_*40%*_(10) = 1.528, *p*_*40%*_ = 0.079 and right targets, *t*_*20%*_(10) = −0.527, *p*_*20%*_ = 0.305; *t*_*40%*_(10) = 1.473, *p*_*40%*_ = 0.086. To test whether our two stop signal ratios affected SSRTs differently for patients from controls, we used difference modified *t*‐tests. We observed no significant differences 20% and 40% SSRTs between I.G. and her controls, *t*(8) = 0.256, *p* = 0.804, or between C.F. and his controls for either left, *t*(10) = 1.121, *p* = 0.289, and right targets, *t*(10) = 1.436, *p* = 0.182. In sum, stop signal ratios did not modulate SSRTs differently in our patients and their respective age‐matched controls.

### 3.3 Control Visually‐Guided Saccade Task

**FIGURE 6:**
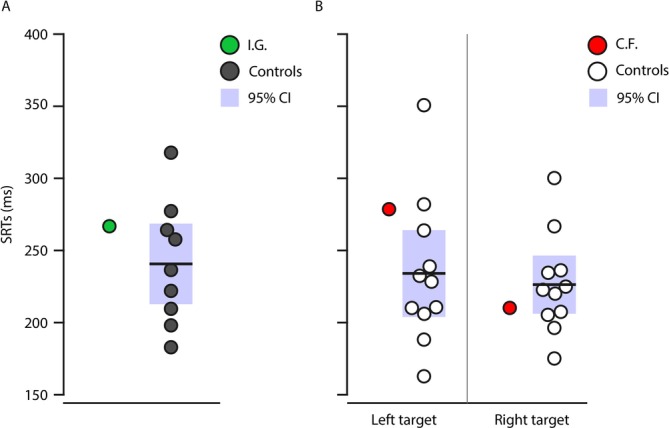
Saccade reaction times for visually guided saccades. In (A), I.G.'s SRTs are presented in green and her controls', in dark grey. In (B), we show C.F.'s SRTs in red and his controls', in white. Shaded areas represent 95% confidence intervals.

We examined SRTs in our patients and controls, in a visually‐guided saccade task requiring no inhibition. Mean SRTs are depicted in Figure 6. Using modified *t*‐tests, we found no significant difference in SRTs for visually‐guided saccades between I.G. and her controls, *t*(8) = 0.580, *p* = 0.289 or between C.F. and his controls for either left, *t*(10) = 0.8178, *p* = 0.216 or right targets, *t*(10) = −0.465, *p* = 0.326.

## 4 Discussion

We investigated the effects of lesions to the dorsal PPC on two distinct inhibition abilities: spatial inhibition, assessed using the IOR task, and response inhibition, measured with the stop signal task. Our results revealed a simple neuropsychological dissociation such that (1) both patients I.G. and C.F. exhibited an absence of the IOR effect in their ataxic hemifield, whereas their respective control groups demonstrated a typical IOR effect; (2) in contrast, their response inhibition performance was not different from that of their control groups for each stop ratio tested separately and, in C.F.'s case, by target side. In a visually‐guided saccade task that did not require either spatial or response inhibition, we found no differences in SRTs between patients and controls. The patients' performance in this control task confirmed that their impairments in the IOR task were not due to general deficits in saccade planning or execution. Taken together, our results point to a specific role of the dorsal PPC in spatial inhibition mechanisms and not in response inhibition.

In the stop signal task, the difference in SSRTs between 20% and 40% was not significant between our patients and their respective control groups, which suggests that the two patients used similar pro‐active inhibition strategies to cancel their motor plan as the control participants. In the IOR task, patient I.G. who had bilateral optic ataxia, showed a reduced IOR effect compared with her controls. Unilateral optic ataxia patient C.F.'s performance was compared both to his controls and between hemifields, where his unaffected hemifield served as his own internal control. Although C.F.'s IOR effect for right targets did not differ from his controls, some facilitation was observed in his left affected hemifield instead of IOR. Together, both patients showed behavior inconsistent with IOR in their affected hemifields compared with their respective controls. This common pattern of results can be attributed to the common lesion of the Brodmann's area 7 in the two patients, suggesting that this neural substrate is crucial for spatial inhibition but not for response inhibition.

Our findings support previous evidence for the role of the dorsal PPC in spatial inhibition. For IOR tasks like ours, which operate within a spatial reference frame, some authors have suggested that the PPC is involved in computing both inhibitory signals of the cue (Sapir et al. 2004) and salience signals (Heinke and Humphreys 2003; Vivas et al. 2003, 2006). The unilateral deficits observed in previous work (i.e., Heinke and Humphreys 2003; Vivas et al. 2003, 2006) and C.F.'s present results may stem from an imbalance of inhibitory signals across internal representations, such as priority maps. Priority maps are map‐like representations where bottom‐up and top‐down processes interact to assign an attentional *priority* to relevant objects. For example, representations of relevant targets are enhanced to facilitate object selection and attention at this location, whereas the representations of irrelevant objects are suppressed to limit their competition for selection (Fecteau and Munoz 2006; Zelinsky and Bisley 2015). In the case of C.F., an imbalance of inhibitory signals within priority maps could then lead to increased weighting of the ipsilesional hemifield relative to the contralesional one, simultaneously biasing target selection and disrupting inhibitory mechanisms in the affected hemifield (Heinke and Humphreys 2003; Vivas et al. 2003, 2006). Taken together, these common findings in two patients suggest that lesions to the dorsal PPC disrupt the encoding of the spatial coordinates of the cue in location‐based IOR.

Although our IOR task results point to specific spatial inhibition deficits, they cannot pinpoint the precise nature of these deficits, but we can speculate on potential explanations. Because IOR relies on finely tuned temporal dynamics, one possibility is that these deficits reflect a disruption in the timing of inhibitory processes. The typical time course of attentional capture and IOR shows that short cue‐target asynchronies (i.e., under 200 ms) produce a facilitation effect at the cued location, whereas IOR emerges between 200 and 300 ms and persists for several seconds (Fecteau and Munoz 2005; Khan et al. 2016; Klein 2000). In our task, we used a fixed cue‐target asynchrony of 500 ms, which we expected to elicit a strong and stable IOR effect. However, it is possible that the temporal dynamics of facilitation and IOR are delayed in our patients. This could explain the facilitation effect found in C.F. and the reduced IOR effect found in I.G. If so, we might observe stronger and stable IOR in our patients at longer cue/target asynchronies than the typical IOR time course described above. This hypothesis is consistent with previous findings showing delayed attentional capture in optic ataxia patients (Gaveau et al. 2008). Alternatively, it is possible that the temporal dynamics of IOR are intact in our patients, and it is instead the spatial dynamics of IOR that are impaired. IOR is expected to be spatially specific to one location. Considering the role of dorsal PPC in oculocentric spatial representations (Bisley and Goldberg 2010; Goldberg et al. 2006), it is possible that lesions in this area disrupt the encoding of the cue or the target location in the oculocentric representation of space in our patients. If the encoding of spatial coordinates is inaccurate, IOR may occur in a different location than the one probed by the target (Sapir et al. 2004). This would be in line with previous work showing oculocentric misrepresentations during arm movements in optic ataxia patients (Blangero et al. 2010; Khan, Pisella, Rossetti, et al. 2005; Khan, Pisella, Vighetto, et al. 2005). A third possibility is that both temporal and spatial dynamics of IOR are impaired in our patients, leading to both delayed and mislocated suppression of the cue‐related activity in a location‐based reference frame. In this case, IOR would occur after 500 ms and at an incorrect, unprobed location. Given our design choices, we cannot differentiate between these possibilities, and we therefore refrain from mechanistic interpretations beyond these speculations. Future research should systematically manipulate both the temporal and spatial components of IOR in optic ataxia patients to clarify the role of the dorsal PPC in building spatial representations for attentional capture and suppression.

Let us revisit previous tentative interpretations of attentional capture deficits in optic ataxia. In a Posner paradigm (Striemer et al. 2007), unilateral optic ataxia patients (including patient C.F.) had slower responses to stimuli presented in their affected hemifield regardless of cueing condition, which was interpreted as reflecting an overall decrease in the salience of stimuli in the ataxic field. When these data were plotted alongside those of Posner and Cohen (1984) on hemineglect (Pisella et al. 2013), it was clear that the deficit to disengage attention from their ipsilesional side was more characteristic of ventral attentional network damage, optic ataxia consecutive to dorsal attentional network damage being rather associated with slow attentional engagement toward the contralesional side. Other studies and reviews have similarly suggested that engagement deficit is more characteristic of damage to the dorsal PPC (Bartolomeo and Chokron 2002; Coll et al. 2024; Gillebert et al. 2011; patients I.G. and C.F., Jurkiewicz et al. 2025; Losier and Klein 2001; Pisella et al. 2013; Martín‐Arévalo et al. 2021; patient I.G., Valdois et al. 2019; Vialatte et al. 2021), affecting localization in the contralesional field, spatial competition between simultaneous stimuli, facilitation of perception and action by endogenous spatial cueing, and reorienting (that requires both disengagement and engagement processes). There might be an interaction between less engagement of attention to the contralesional cue in optic ataxia facilitating inhibition processes. Our present findings about spatial inhibition, alongside our previous results on anti‐saccades in optic ataxia (Ouerfelli‐Ethier et al. 2021, 2023), instead revealed deficits to inhibit contralesional locations previously attended in space. As for response inhibition, one can also consider that, once planned, the motor response is independent from the visual target saliency and its localization. Nevertheless, if the initiation of the motor response toward the contralesional side requires more effort, this would lead to make a prediction of fewer stop errors in the ataxic visual field, which was indeed observed as a numerical trend in the present study but did not reach significance. However, the relationship between engagement and inhibition remains to be directly assessed with paradigms better suited for attentional capture than saccadic IOR.

In contrast to spatial inhibition, our results point to an unnecessary role of the dorsal PPC in response inhibition. Specifically, I.G. and C.F. did not show impaired stop signal performance compared with their respective controls. This reinforces the notion that the anti‐saccade deficits in optic ataxia patients found in previous studies resulted from impaired spatial, rather than response inhibition mechanisms (Ouerfelli‐Ethier et al. 2021, 2023). The role of the dorsal PPC in such response inhibition tasks hinted at in previous imaging (Congdon et al. 2010; Hu and Li 2012) and TMS (Osada et al. 2019) studies may thus be related to non‐inhibitory mechanisms, such as the visual representation of the target (Paré and Wurtz 2001), and spatial computation of the saccade goal location (Ouerfelli‐Ethier et al. 2021, 2023). The interconnections between the parietal and frontal cortex could also explain the previously reported PPC activity during response inhibition (Friedman‐Hill et al. 2003; Osada et al. 2019). In this case, the activity observed in the PPC in previous studies would be a by‐product of or a relay for frontal cortex activation, an area known to be crucial for response inhibition (Aron et al. 2003, 2014; Aron and Verbruggen 2008; Boehler et al. 2010; W. Cai et al. 2011; Hampshire et al. 2010; Lee and Hsieh 2017; Munakata et al. 2011; Swick et al. 2008; Zhang and Li 2012). Double‐dissociation paradigms with both patients with PPC lesions and patients with frontal lesions could further clarify the specific contributions of frontal and parietal areas to spatial, response inhibition, and the non‐spatial components of response inhibition. In summary, our results complement previous work outlining an adjacent role of the dorsal PPC in response inhibition tasks while further highlighting the need to clarify its role in the non‐inhibitory processes involved in response inhibition.

Preserved response inhibition combined with impaired IOR in our optic ataxia patients is thus consistent with independent spatial and response inhibition mechanisms. The stop signal task is not spatial‐specific, the way IOR is. Past literature has shown that SSRTs estimations are independent of the spatial location of the cue and its modality (Asrress and Carpenter 2001; Logan 1981; van den Wildenberg and van der Molen 2004), further highlighting the spatial invariance of the stop signal task, and its appropriateness to investigate purely response inhibition processes. In contrast, proposed mechanisms of saccadic IOR have often emphasized motor response selection, suggesting that cue presentation triggers a counter‐motor plan to inhibit the initial motor planning toward the cue (Ro et al. 2003). Similarly, it has been suggested that onset of the target following the flashed cue resets the threshold of the saccadic system for movement execution (Hafed and Ignashchenkova 2013; Salinas and Stanford 2013). This reset is thought to involve “countermanding” or response inhibition processes, where the target onset functions as a stop signal to interrupt the ongoing motor program directed toward the previously cued location (Hafed and Ignashchenkova 2013). The dissociative nature of our findings in our patients do not support this view. If response inhibition subtended IOR processes, we would have also observed impaired response inhibition in our patients as measured by our stop signal task. We thus propose that purely spatial mechanisms underly saccadic IOR, and the inhibition of the saccade movement (i.e., response inhibition) to the cue location may be instead, simply saliency suppression at this location. This suppression would counteract the attentional capture or reactivation of the cue location once the target appears at the same location, leading to the increased SRTs observed in our controls. In contrast, in our patients, the lingering suppression at the cue location during target presentation may be impaired, delayed or mislocated, as discussed earlier, which would account for a lack of IOR in their ataxic hemifield. In other words, our findings suggest that IOR is driven by spatially selective inhibition processes (i.e., suppression and reactivation), rather than by the inhibition of motor plans.

Further, the independence of spatial and response inhibition mechanisms suggested by our findings may mirror the segregation of neuronal populations involved in visual location‐based and motor planning throughout the cortex (Brown et al. 2008; Juan et al. 2004; Ray et al. 2009; Thompson et al. 1997; Thompson and Bichot 2005; Wardak et al. 2011). Spatial inhibition and spatial competition have been mainly associated with the frontoparietal network (Jerde et al. 2012; Lanssens et al. 2020; Ptak 2012; Wardak et al. 2011). This network plays a particular role in the inhibition of irrelevant stimuli which compete for attention (Ferrante et al. 2018; Friedman‐Hill et al. 2003; Molenberghs et al. 2007) via sensory‐gating mechanisms (Chelazzi et al. 2019; Pascucci et al. 2018; Popov et al. 2017). The frontoparietal network has also been shown to subtend the inhibitory processes involved in IOR (Bourgeois et al. 2013a, 2013b; Seidel Malkinson and Bartolomeo 2018). In contrast, response inhibition has been shown to rely on cortico‐basal ganglia pathways involving the frontal cortex (Aron and Poldrack 2006; Casey et al. 1997; Ghahremani et al. 2012; Nambu et al. 2002, 2003; Purves et al. 2018; Rieger et al. 2003). Research on Parkinson's disease suggested that there may be no lateralisation in the processes of motor inhibition at the level of the basal ganglia (Mirabella et al. 2017), supporting a global low‐level process operating at the level of saccade execution, irrespective of its direction. Our results thus highlight the involvement of different neural pathways in spatial‐ and motor‐based inhibition, but further neurophysiological, neuroimaging or clinical studies are needed to clarify the respective circuits involved in both types of inhibition.

A limitation to our current findings is the unbalanced number of trials across the four potential target locations in the IOR task. This was due to our patients' scotoma, particularly C.F.'s which was discovered accidently during testing. We attempted to limit different intra and inter hemifield effects in our unilateral patient by removing trials, ensuring balanced trials across locations. We acknowledge that our analyses should be interpreted with caution considering the small number of trials. Furthermore, potentially different cue‐target inhibitory interactions within versus across hemifields such as reported previously (Ouerfelli‐Ethier et al. 2021) could not be addressed in the present study.

## 5 Conclusions

Overall, we observed selective impairments in spatial inhibitory processes in our patients during the IOR task. In contrast, response inhibition abilities, as measured by the stop signal task, were preserved. This simple dissociation suggests that spatial and motor‐based inhibitory mechanisms rely on distinct neural substrates. Specifically, the dorsal PPC appears to play a critical role in spatial inhibition, whereas motor‐based inhibition may involve different brain regions. These findings underscore the need for future research to disentangle the neural correlates of distinct inhibitory mechanisms, particularly in clinical populations with impaired inhibition abilities, such as frontal lesioned patients.

## Author Contributions

**Julie Ouerfelli‐Ethier:** conceptualization, data curation, formal analysis, funding acquisition, investigation, methodology, project administration, visualization, writing – original draft, writing – review and editing. **Tristan Jurkiewicz:** data curation, investigation, methodology, project administration, writing – review and editing. **Isabella Comtois‐Bona:** investigation, project administration. **Thomas Carrier:** investigation, project administration. **Aarlenne Z. Khan:** conceptualization, formal analysis, funding acquisition, investigation, methodology, resources, supervision, writing – review and editing. **Laure Pisella:** conceptualization, formal analysis, funding acquisition, methodology, resources, supervision, writing – review and editing.

## Funding

This work was supported by the Natural Sciences and Engineering Research Council of Canada (BESD‐546502‐2020), Études Supérieures et Post‐doctorales and École d'optométrie merit scholarship, Canada Research Chairs, the Université de Lyon Idex Mobility fund, the Réseau de la santé de la vision du Québec, and the Centre National de la Recherche Scientifique.

## Conflicts of Interest

The authors declare no conflicts of interest.

## Data Availability

The data and analysis scripts required to reproduce the results and figures are available on the Open Science Framework repository at the following link: https://doi.org/10.17605/OSF.IO/BNH9E.
